# Ecosystem Microbiology of Coral Reefs: Linking Genomic, Metabolomic, and Biogeochemical Dynamics from Animal Symbioses to Reefscape Processes

**DOI:** 10.1128/mSystems.00162-17

**Published:** 2018-03-13

**Authors:** Linda Wegley Kelly, Andreas F. Haas, Craig E. Nelson

**Affiliations:** a Department of Biology, San Diego State University, San Diego, California, USA; b Department of Marine Microbiology and Biogeochemistry, Royal NIOZ, the Netherlands Institute for Sea Research in cooperation with Utrecht University, Texel, Netherlands; c Center for Microbial Oceanography: Research and Education, Department of Oceanography and Sea Grant College Program, University of Hawai‘i at Mānoa, Honolulu, Hawai‘i, USA

**Keywords:** biogeochemistry, coral reef, ecosystem, metabolomics, metagenomics

## Abstract

Over the past 2 decades, molecular techniques have established the critical role of both free-living and host-associated microbial partnerships in the environment. Advancing research to link microbial community dynamics simultaneously to host physiology and ecosystem biogeochemistry is required to broaden our understanding of the ecological roles of environmental microbes. Studies on coral reefs are actively integrating these data streams at multiple levels, from the symbiotic habitat of the coral holobiont to microbially mediated interactions between corals and algae to the effects of these interactions on the microbial community structure, metabolism, and organic geochemistry of the reef ecosystem. Coral reefs endure multiple anthropogenic impacts, including pollution, overfishing, and global change. In this context, we must develop *ecosystem microbiology* with an eye to providing managers with microbial indicators of reef ecosystem processes, coral health, and resilience to both local and global stressors.

## Abstract (precis)

Over the past 2 decades, molecular techniques have established the critical role of both free-living and host-associated microbial partnerships in the environment. Advancing research to link microbial community dynamics simultaneously to host physiology and ecosystem biogeochemistry is required to broaden our understanding of the ecological roles of environmental microbes.

## CORAL REEFS AS RAPIDLY CHANGING MICROBIAL ECOSYSTEMS

Coral reefs present a rich opportunity to develop a holistic microbial system biology integrating across scales from evolutionarily entrenched animal symbioses to the free-market synchrony of the oceanic plankton. As ecological systems, coral reefs maintain remarkably high rates of biomass production even as they emerge from some of the most nutrient-depleted oceans on Earth. Scleractinian corals, the keystone animals that define the shape and extent of reefs, maintain stable mutualisms with zooxanthellae (photosynthetic dinoflagellates in the genus *Symbiodinium*) and a complex consortium of bacteria, archaea, fungi, and viruses, collectively referred to as the coral holobiont. These microbial symbionts mediate the stringent recycling of nutrients that establish corals as the dominant competitors in reef habitats. Threatened on all sides by the upheaval of the Anthropocene epoch, reefs worldwide are undergoing rapid change. This includes shifts to fleshy algal dominance as overfishing and nutrient enrichment foster algal growth and multiple stressors both local (sedimentation, pollution) and global (warming, acidification) weaken corals. Because of the central role of microbial communities in reefs across water, sediment, corals, and a host of other benthic and pelagic denizens, it is apparent that microbial ecosystem science will be key to understanding how, when, and where reefs are most resilient to this onslaught.

## ECOSYSTEM MICROBIOLOGY SPANS SYMBIOSIS TO BIOGEOCHEMISTRY

**FIG 1:**
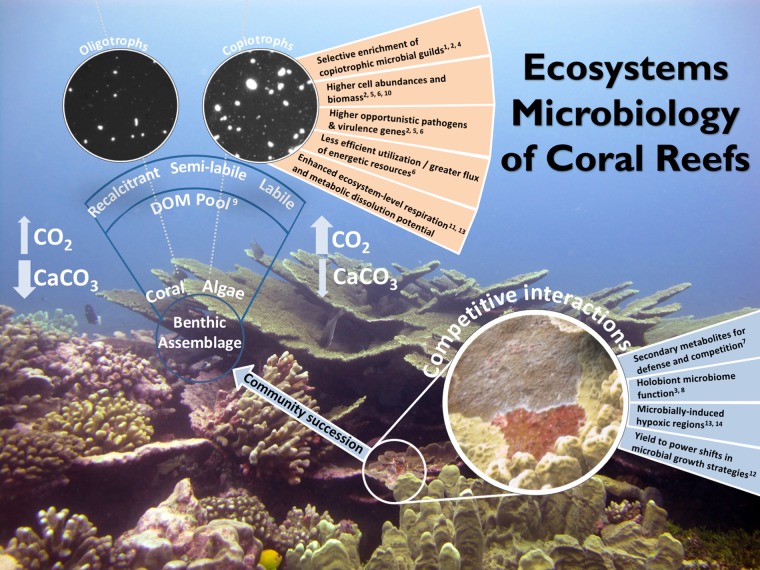
Ecosystem microbiology embraces a holistic characterization of the role of microbes in coral reefs from molecules to physiology to biogeochemical processes. These strategies will provide a mechanistic understanding of what governs macroorganismal community shifts and how this benthic community succession alters ecosystem function.

Driven by the need to understand reef transitions on a global scale, we posit that research actively linking microbial community dynamics simultaneously to host physiology and biogeochemical transformations will unravel the mechanistic linkages underpinning whether coral reefs live or die. Most of the microbial taxa inhabiting marine systems are poorly characterized, with limited representation via culturable isolates to model adaptive physiology and functional attributes. As a result, the toolbox of methods we and others use to investigate these systems is dominated by modern molecular approaches, including high-throughput sequencing of phylogenetic marker genes to define community structure (1, 2) and comparative shotgun metagenomics and metatranscriptomics to resolve dynamics in community function (3–6). By explicitly considering host-associated microbiomes as drivers of environmental interactions, microbial composition and metabolic function across hosts and habitats can be used to contextualize the magnitude and direction of microbial influences on reef organismal and ecosystem processes. Further compositional descriptions can be employed using untargeted metabolomics of both host tissue (7, 8) and the overlying water column (2, 9) to characterize biochemical products exuded by benthic producers and subsequent utilization by heterotrophic microbes (2, 10–12). Finally, measurements of bulk biogeochemical fluxes between hosts and the surrounding environment (1, 11, 13) can estimate to what degree macroorganismal community shifts translate to microbial responses that alter basic ecosystem properties such as primary production, respiration, and food web structure. Utilizing ecosystem microbiology, the field will move from broad descriptive comparisons of genomic and metabolomic data sets toward synthesizing information across methods and scales with rapidly advancing statistical, bioinformatic, and data visualization approaches to provide a holistic view of microbial interactions in the environment (Fig. 1). This advancement in microbial system science will help build a predictive framework to assess responses to local disturbances and global change while simultaneously providing a more mechanistic understanding of reef ecosystem function.

## REEF EXOMETABOLOMES DRIVE MICROBIAL POPULATION AND METAGENOMIC STRUCTURE

The oceanic interface of reefs, as a blossoming wall of macroorganismal diversity and biomass bathed in waters dominated by submicrometer bacteria and archaea, forms a striking gradient that defines the microbial system biology of the coral ecosystem. Reefs maintain their own unique planktonic microbiomes and geochemical milieu even when flushed continually by the surrounding ocean (1). Each of the dominant reef benthic primary producers we have studied releases upwards of 10% of its daily photosynthesis as dissolved organic matter (DOM) (10, 11). The composition of these exudates is determined by the source species (2), and defines a significant portion of the reef exometabolome. Both host-associated and planktonic microbes decompose available metabolites (2, 10–13), utilizing energy captured during the oxidation of these organic substrates, which is critical to nutrient cycling in these oligotrophic marine ecosystems. These organic metabolites derived from benthic communities persistently induce rapid microbial growth at concentrations as low as 1 ppb, eliciting predictable patterns in bacterioplankton community structure that can be distinguished in both culture incubations and across *in situ* communities on reefs varying in benthic composition (2, 4, 6, 10). Both the genotype of zooxanthellae and the relative abundances of specific bacteria and archaea influence the metabolome of coral holobiont tissues (8), as do local competitive interactions with algae (7). Given the significant role of benthic exometabolites in determining the structure and function of reef microbes, a primary research focus in the next decade must be classification of the overwhelming diversity of compounds comprising marine DOM. Efforts to implement nontargeted molecular characterization of DOM by high-resolution liquid chromatography-tandem mass spectrometry (9) will facilitate the discovery of exometabolites produced in response to competitive interactions or those released into the environment and how the subsequent flux of specific classes of compounds influences microbial growth and respiration.

## CONNECTING THE MICROBIOME AND METABOLOME OF THE CORAL HOLOBIONT TO REEF INTERACTIONS

Coral holobionts exert a significant influence over reef metabolism and geochemistry through photosynthesis, nitrogen fixation, calcification, and dissolution; thus, the influence of holobiont microorganisms on these ecosystem processes is also of interest. The role of this microbiome in coral health and resistance to stressors remains a central question in coral reef biology. While past characterization of this complex coral microbiome has focused on marker gene surveys, whole-community shotgun sequencing is required to provide functional information about the metabolic capacity of coral microbiomes (3); in the coming decade, advancing sequencing and bioinformatic tools from disparate disciplines will allow investigators to circumvent the significant molecular signal from the host and zooxanthellae genomes. Beyond the symbiotic microbiome, the interfaces between distinct coral and algal holobionts represent the battlefront of fierce competition for space on the reef: Winning or losing at these interaction interfaces determines the benthic community structure on reefs. These zones exhibit altered diel oxygen dynamics, with a drastically increased nighttime oxygen demand that often reaches hypoxic levels known to harm corals (13, 14). These interactions enhance heterotrophic microbial activity favoring faster, less efficient metabolic strategies (12), emphasizing the potential for host microbiome interactions to foster biogeochemically relevant features such as anaerobic microsites. Surveys conducted across the Pacific demonstrate that corals adjacent to algae also underwent shifts in their metabolomic signatures to exhibit competitive, interaction-specific compounds that differed significantly from those associated with interactions with other corals (7). Because organic exudates from algae have been shown to induce coral death (5) and select for potential coral pathogens (2), resolving the connection between coral and algal microbiome structure and metabolomic outcome will be crucial in determining the mechanisms underlying algal phase shifts.

## REEF MICROBIALIZATION CAUSES SHIFTS IN REEF BIOGEOCHEMICAL PROCESSES

The changes in reef microbial community structure associated with algal phase shifts can have significant impacts on reef biogeochemistry. For example, mesocosms deployed *in situ* on reefs demonstrate how benthic macroalgae can enhance microbial growth and organic matter remineralization relative to coral-dominated benthos (11). Microbial growth strategies are highly dependent on the amount of energy captured during the oxidation of different organic compounds, such that a shift in the oxidation state of compounds released by different benthic producers alters both the bioavailability of exometabolites and the potential energy gained during remineralization. A surplus of bioavailable energy in the exometabolite pool dominating algal reefs favors opportunistic, fast-growing microbes that inefficiently deplete organic resources, reducing concomitant rates of trophic transfer. This process of microbialization can start at the scale of coral-algal interactions: the inefficient heterotrophic microbial activity at coral-algae interfaces (12) creates anoxic zones (13) that harm corals (14), as reflected in coral transcripts and metabolites (7), fundamentally reducing coral productivity. Microbialization is induced as algal exudates alter both organic matter composition and water column microbial community structure to depress the efficiency of carbon biomass conversion (2, 10). The copiotrophic microbial taxa enriched by the exometabolome of degraded reefs reduce the efficient remineralization of organic matter central to the productivity of reef ecosystems (5, 6, 12). This new microbial community structure is enriched in virulence factors and opportunistic pathogens that may further stress corals (2, 4, 5). Our recent global survey demonstrated that alga-dominated reefs have less DOM, more microbial biomass, and a shift in microbial carbon metabolism pathways to favor inefficient biomass conversion, consistent with the predictions of microbialization (6). Thus, shifts in the type of reef macroorganisms as have been observed worldwide can fundamentally alter the recycling of carbon in reef ecosystems, with implications for transfer to higher trophic levels.

## ESTABLISHING HOLISTIC MONITORING TOWARD PREDICTION OF REEF MICROBIALIZATION, CORAL STRESS, AND RESILIENCE

Linking community genomics and transcriptomics to metabolomics within hosts, between holobionts, and with the surrounding planktonic microbiome will go a long way toward resolving central questions in coral reef ecology by defining how stresses to individual reef organisms translate through host and habitat microbiomes to wholesale shifts in reef geochemistry. However, we envision ecosystem microbiology moving toward a predictive understanding of microbial community function and dynamics (15), requiring that we continue to emphasize more quantitative process measurements, including microbial population abundance and growth dynamics, metabolite production and utilization rates and pathways, and measurements of ecosystem biogeochemical fluxes using coupled manipulative experiments and *in situ* surveys. The potential for using microbial taxa and metabolites as pragmatic windows into the physiology, stress response, and resilience of corals and coral reefs is of great interest to coral biologists, reef ecologists, and those tasked with managing our coral reefs for the future. To facilitate the development of microbial and molecular biomarkers of reef health and resilience, we are establishing baselines of these properties across dozens of Pacific islands and atolls; combining broad regional surveys and detailed experimentation will enable the detection of shifts in ecosystem functioning and define the chemical drivers and microbially mediated mechanisms maintaining the health of reefs worldwide.
